# Explanatory Judgment, Moral Offense and Value-Free Science

**DOI:** 10.1007/s13164-015-0282-z

**Published:** 2015-08-16

**Authors:** Matteo Colombo, Leandra Bucher, Yoel Inbar

**Affiliations:** 1 Tilburg Center for Logic, Ethics and Philosophy of Science, Tilburg University, P.O. Box 90153, 5000 LE Tilburg, The Netherlands; 2 Department of Psychology and Cognitive Sciences, Justus Liebig Universität Gießen, Gießen, Germany; 3 Department of Social Psychology, University of Toronto, Toronto, ON Canada

## Abstract

A popular view in philosophy of science contends that scientific reasoning is objective to the extent that the appraisal of scientific hypotheses is not influenced by moral, political, economic, or social values, but only by the available evidence. A large body of results in the psychology of motivated-reasoning has put pressure on the empirical adequacy of this view. The present study extends this body of results by providing direct evidence that the moral offensiveness of a scientific hypothesis biases explanatory judgment along several dimensions, even when prior credence in the hypothesis is controlled for. Furthermore, it is shown that this bias is insensitive to an economic incentive to be accurate in the evaluation of the evidence. These results contribute to call into question the attainability of the ideal of a value-free science.

## Introduction

Imagine that you are a member of an advising panel, where your task is to assess the quality of a number of empirical studies. One study provides evidence that being raised by a same-sex couple increases the chances of suffering from certain developmental disorders. This hypothesis does not apparently carry any normative content and can be objectively tested, yet you may find it morally offensive, because some of your stereotypes and prejudices may be triggered upon reading about it. You are aware of these facts, and yet remain convinced that your personal values will not influence your appraisal of the quality of the evidence gathered for the hypothesis. You are confident, furthermore, that the fact that you have a monetary incentive to properly assess how the evidence bears on the hypothesis will not make any significant difference in your considered judgment either. But will these convictions of yours be borne out in practice? Will your personal values play no significant role in your assessment of the evidence?

The conviction that your appraisal of a scientific study will remain unaffected in the situation just described is consistent with a traditional view in philosophy of science that relies on the distinction between epistemic and non-epistemic values (Kuhn 1977; Laudan 1984). According to this view, the assessment of how evidence bears on scientific hypotheses should be directed by epistemic values. Your judgments about what the evidence is for a certain hypothesis and how strong the evidence supports the hypothesis should be affected only by truth-conducive, epistemic values, such as confirmation, empirical accuracy, and predictive and explanatory power.

Non-epistemic values, including moral and economic values, may enter into other stages of scientific practice (Machamer and Wolters 2004). Non-epistemic values may influence scientists’ choices about which research questions to work on; they may affect policy-makers’ decisions about how scientific results are to be used; and they may impact funding agencies’ judgments about which research projects deserve financial support. But, as maintained by many philosophers of science, the influence of non-epistemic values on these choices “is clearly *not* sufficient, by itself, to deprive the social or the natural sciences of their value-free character from a *cognitive* point of view” (Dorato 2004, p. 56; see Longino 1996 for a criticism of the epistemic vs. non-epistemic value dichotomy).

According to the ideal of the value-free character of science, scientists should strive to minimize the influence of non-epistemic values on their assessment of the evidence for a hypothesis; in particular, “propositions about what states of affairs are desirable or deplorable could never be evidence that things are, or are not, so” (Haack 1993, p. 35). Although non-epistemic values “may shape scientific knowledge to the extent that they play a role in the definition of research programs, in the choice of questions deemed scientifically interesting, in the way scientific results might be applied etc., this contextualization of the *goals* of science does not in itself threaten objectivity. More epistemologically challenging is the distinct charge that the very *content* of scientific knowledge is shaped by contextual values” (Ruphy 2006, pp. 189–90). In essence, the idea is that scientific reasoning is objective to the extent that the appraisal of scientific hypotheses, which contributes to producing scientific knowledge, is not influenced by non-epistemic values, but only by the available evidence.

This idea can be better qualified, after we reflect that the evidence relation is a three-place relation that connects data, hypotheses and background knowledge. If one’s background knowledge includes information about the moral consequences of believing that a hypothesis is true (or is not true), then non-epistemic values can sometimes affect one’s assessment of the evidence for that hypothesis (Sober 2007). For instance, the non-moral proposition “Drug X is safe.” is evidentially related to the moral proposition “Good consequences accrue to the patients.” when it is also believed that the patients’ physician is competent and well meaning. In cases like this one, background knowledge allows for moral and non-moral propositions to be evidentially related (i.e., one raises the probability of the other).

Background knowledge also allows for the non-epistemic moral values that determine the expected utility of believing that a certain hypothesis is true (e.g., believing that drug X is safe) to provide a non-trivial lower or upper bound on the probability that the hypothesis is true (e.g., that drug X is safe) (Sober 2007, pp. 114–5). But, in this type of case, we should already possess information about the probability that the hypothesis is true in order to answer the question of whether believing the hypothesis has better moral consequences (i.e., higher expected utility) than not believing it. So, “judgments about the ethical consequences of believing a proposition cannot supply *new* information about the probability of the proposition” (Sober 2007, p. 117). The fact that believing a certain hypothesis has good or bad moral, social or political consequences cannot provide *new* evidence for or against the hypothesis’s being true.

The ideal of value-free science and objectivity in scientific reasoning can then be reformulated as the idea that non-epistemic values should not affect the appraisal of the relation between hypothesis, data, and background knowledge. In particular, moral information should not affect the assessment of the evidence available for a hypothesis *over and above* the hypothesis’s prior credibility.

Although the value-free ideal makes a normative claim, one obvious question is whether this normative ideal is actually attainable. In recent literature in philosophy, history and sociology of science, it has been argued that both epistemic *and* non-epistemic values are crucial for assessing what counts as sufficient evidence, and that social, political and economic structures influence the practice of science (e.g., Douglas 2009; Elliott 2011; Longino 1990; Reiss and Sprenger 2014, Sec 3; Resnik 2007).

Less attention has been paid to the psychology of scientific reasoning, and in particular to the psychological attainability of objective, value-free scientific reasoning. This is surprising, since *reasoning* and *valuing* are obviously psychological processes.

If reasoners systematically use moral values to gain new information about the probability of a given scientific hypothesis, then that would be strong evidence that directly speaks against the attainability of the value-free ideal. Insisting that science should be free of non-epistemic values, when human reasoners cannot achieve value-freedom, would perpetuate a myth “that interferes with the public’s understanding of the scientific process and may, paradoxically, undermine the public’s trust in science” (Elliott and Resnik 2014, p. 648).

While the social and institutional character of science might compensate for some of the effects of moral values on scientific reasoning (e.g., Longino 1990; Popper 1934), it would make little sense to calling for scientists to even try to achieve the value-free ideal. Instead, it may be more important to investigate the psychological mechanisms of scientific reasoning, and examine more closely what kinds of social and institutional settings promote sound scientific reasoning.

Questions about the psychological attainability of objectivity in scientific reasoning are not new. In his 1620 *Novum Organum*, Francis Bacon already recognized that scientific reasoning can be systematically misled by several kinds of “idols.” In particular, Bacon foreshadowed that motivational factors that have little to do with epistemic value and objectivity are powerful determinants of scientific reasoning and judgment. Writes Bacon: “the human understanding resembles not a *dry light*, but admits a tincture of the will and passions, which generate their own system accordingly: for man always believes more readily that which he prefers” (Sect. 49). Bacon’s contention has been substantiated by a large body of empirical results in psychology (see e.g., Kunda 1990), which has been hardly discussed in philosophy of science in relation to the attainability of the value-free ideal.

One finding particularly relevant to these debates is that reasoning processes are influenced by several motivational factors. Two kinds of motivational factors are associated with accuracy goals and directional goals. *Accuracy goals* motivate reasoners to “arrive at an accurate conclusion, whatever it may be,” whereas *directional goals* motivate them to “arrive at a particular, directional conclusion” regardless of its accuracy (Kunda 1990, p. 480). Both kinds of motivations have been found to affect reasoning and judgment in a variety of tasks.

For example, Lord et al. (1979) famously provided evidence that people tend to interpret ambiguous scientific evidence as supporting their favored conclusion. In their study, participants were presented with two mock scientific reports concerning the effectiveness of death penalty in deterring crime. While one report provided supporting evidence for the deterrent efficacy of the death penalty, the other provided disconfirming evidence. Participants’ prior convictions about death penalty were found to predict their explanatory judgments. Both proponents and opponents of capital punishment rated the report that agreed with their prior convictions as more convincing, and were more adept at finding flaws in the report that disagreed with their prior convictions. As a result, the mixed evidence from the two reports led participants to become even more certain of their pre-existing beliefs regarding the efficacy of capital punishment.

Along the same lines, it has been shown that both scientists’ and laypeople’s explanatory judgments about the quality of the results and methodological soundness of a piece of experimental research are predicted by their prior beliefs. Experimental research is rated as higher-quality and methodologically sound, when the experimental results conform to their prior beliefs (Koehler 1993; Greenhoot et al. 2004).

Further experimental results have confirmed that judgment about scientific evidence is often biased in subtle and intricate ways (MacCoun 1998). We tend to assess scientific reports about the validity of a psychological test as more or less reliable as a function of our (good or bad) performance on the test (Wyer and Frey 1983; Pyszczynski et al. 1985). We disbelieve alleged medical evidence that suggests that certain behaviour has negative health consequences, if we routinely engage in that behaviour (Kunda 1987). We often employ less rigorous standards of assessment for information that favors our preferred conclusions than for information that we find undesirable (Ditto and Lopez 1992). More generally, motivational states that have no obvious epistemic value can influence many of our beliefs about the world (e.g., Norton et al. 2004; Uhlmann and Cohen 2005; Balcetis and Dunning 2006; Harris et al. 2009; Lewandowsky et al. 2013; see also Krizan and Windschitl 2007 for an evaluation of the literature on the *desirability bias*).

These findings suggest that the evaluation of scientific evidence may be biased by the extent to which its conclusions are found desirable. However, these studies provide weak support for the claim that non-epistemic values systematically affect the appraisal of the relation between a scientific hypothesis, data, and background knowledge, because they did not control for hypotheses’ prior credibility and did not assess the extent to which accuracy incentives can mitigate the effect of directional goals. So, they do not speak directly as to what extent the perceived moral offensiveness of a scientific hypothesis can bias one’s assessment of the evidence available for the hypothesis *over and above* the hypothesis’s prior credibility.

It may be supposed that it is unlikely that the experimental participants in at least some of the studies reviewed above had any prior opinion relevant to the task or needed any special incentive to be accurate; and so it may be supposed that it would have been superfluous to control for hypotheses’ prior credibility or to manipulate accuracy goals. This supposition might be correct, yet the available empirical evidence does not support it.

In what follows, we report two studies where we asked exactly how the prior credibility of scientific hypotheses, their perceived moral offensiveness, and the motivation to be accurate in judging their explanatory power affect one’s assessment of putative scientific reports. We controlled for the prior credibility of the hypotheses contained in the scientific reports, and we manipulated accuracy incentives. Thus, our studies contribute to advance current literature in the philosophy and psychology of scientific reasoning by providing strong evidence that non-epistemic values systematically and robustly affect the appraisal of the relation between hypothesis, data, and background knowledge.

Specifically, Study 1 tested whether differences in scientific hypotheses’ perceived moral offensiveness predict differences in explanatory judgments, even when the prior credibility of the hypotheses is controlled for. Study 2 tested whether a monetary incentive to be accurate in the assessment of the evidence has a mitigating effect on the impact of the perceived moral offensiveness of a hypothesis on explanatory judgments about the hypothesis.

Overall, our results show that explanatory judgments about a scientific hypothesis are robustly sensitive to the perceived moral offensiveness of the hypothesis. This finding directly supports the idea that one’s assessment of the evidence in support of a scientific hypothesis can be systematically affected by judgments about the moral value of the hypothesis, which suggests that scientific reasoning is imbued with non-epistemic values.

The rest of the paper is structured as follows. Section 2 describes a preliminary test we ran on the experimental material that was used in our two studies. Section 3 and Section 4 present our two studies. Section 5 puts the results into a broader philosophical perspective and discusses their implications for the psychology of explanatory reasoning and for the ideal of a value-free science. The Conclusion summarises our contribution to current literature and traces three avenues for further research.

## Test of Material: Finding Scientific Hypotheses That are Morally Offensive

Prior to the experimental studies, a test of material was performed in order to find the most suitable hypotheses for our main experiments. The aim was to obtain an estimate about the degree to which certain hypotheses were judged as “morally offensive” and as “credible.”

### Participants

Forty undergraduates from the Tilburg University in The Netherlands (mean age 23.84 years, *SD* 5.52; 22 male), all native speakers of Dutch took part in the pre-study. All participants gave their informed written consent for participation in the study, in accordance with the Declaration of Helsinki. In particular, participants received careful debriefing about the potential offensiveness of the study material.

### Materials and Procedure

Participants were presented with booklets, each page of which contained one statement (in Dutch) of a scientifically testable hypothesis, for a total of 18 statements (see Appendix 1). Statements were presented in a different and random order to each participant. After reading a statement, participants were asked two questions: “Do you find this statement morally offensive?” and “Do you find this statement credible?”

Judgments about both the credibility and moral offensiveness of the statements were collected on a 5-point scale, ranging from “Not at all” to “Very much”.

### Results

Based on the mean ratings we obtained, the statements were grouped into *morally offensive* (mean rating for moral offensiveness ≥3.5) and *morally neutral* items (mean rating for moral offensiveness <2). Our criteria for selecting suitable items for studies 1 and 2 were twofold. On the one hand, suitable items should be matched for their prior credibility. On the other hand, half of the items should be morally offensive, while the other half morally neutral so that they could be used as control items in our two main studies.

Using these criteria, seven items were selected: all but one of these seven items received a mean credibility rating <2; three items were *morally neutral*, four items were *morally offensive*. Table 1 shows mean ratings of morality and credibility, as well as correlations of the ratings.

**Table 1:** Mean ratings of morality and credibility, *M* (standard deviations, *SD*) and correlations, *r,* between morality and credibility

Statement	Used in	Mean moral ratings^a^ *M* (*SD*)	Mean credibility ratings^a^ *M* (*SD)*	Correlation between moral and credibility ratings (Spearman’s rho) *r*
Eating pizza regularly every day increases the chances of immunity to flu.	Study 1	*M* = 1.85 *(SD = 1.27*)	*M* = 1.33 (*SD = 0.53*)	n.s. (not significant)
Consuming breakfast regularly every morning increases the chances of having a healthy body mass index	Study 1 and 2	*M* = 1.23 (*SD = 0.48*)	*M* = 3.85 (*SD = 0.89*)	n.s.
Attending religious service frequently, at least once a week, increases the chances of maintaining a good health.	Study 1 and 2	*M* = 1.18 (*SD = 1.22*)	*M* = 2.05 (*SD = 1.06*)	n.s.
Men are more successful than women because they are more motivated and they have more cognitive capacities.	Study 1 and 2	*M* = 3.70 (*SD = 1.18*)	*M* = 1.80 (*SD = 0.82*)	*r* = .382; *p* = .015
Developmental disorders occurs more often in children with parents of the same gender.	Study 1 and 2	*M* = 3.47 (*SD = 1.38*)	*M* = 1.85 (*SD = 0.77*)	*r* = −.366; *p* = .020
Long sleepers have a bigger chance of being engaged in sexual abuse than children.	Study 1 and 2	*M* = 3.5 (*SD* = *1.41*)	*M* = 1.30 (*SD* = *0.56*)	n.s.
Being infected with the Merrosa-virus increases the chances of being homosexual in mammals.	Study 2	*M* = 4.20 (*SD* = *1.14*)	*M* = 1.15 (*SD* = *0.36*)	n.s.

^a^Ratings: 1 = Not at all; 2 = Slightly; 3 = Somewhat; 4 = Quite a bit; 5 = Very much

## Study 1: Moral Offense and Explanatory Judgment

### Participants

One hundred and one participants took part in Study 1. 44 female and 57 male participants with a mean age of 32 years (std = 9.92; 52 native speakers of English, 49 other languages,^1^completed the experiment for a small monetary payment. Participants received careful debriefing in the instruction of the experiment, prior to the exposure to the experimental material, informing them about the potential offensive character of the material’s content. The fictitious character of the short reports was particularly emphasized after completion of the experiment.

### Materials and Procedure

The experiment was conducted as an online experiment in English language, using Amazon Mechanical Turk. Participants read six short reports about alleged scientific studies (Appendix 2). The reports were presented in a different, random order to each participant. Each alleged study provided evidence for one of the hypotheses, which were selected based on the results of the pre-study: hence, three of the six scientific reports provided evidence for morally offensive hypotheses; the other three provided evidence for morally-neutral hypotheses.

Participants were asked to carefully assess each scientific report along five dimensions. Specifically, they made judgments about the extent to which “This report is plausible;” “The research described in the report is convincing;” “This research should be funded;” “This study is well-conducted;” “This study provides strong evidence for the conclusion.”^2^ Participants’ judgments were collected on 5-point scales with the extremes “I strongly disagree” “I strongly agree”—an “I don’t know” option could also be selected.

### Results

Ratings on the five dimensions (“This report is plausible”, “This research is convincing”, “This research should be funded”, “This study is well conducted”, “This study provides good evidence regarding the conclusion”) of the morally offensive studies were compared to morally neutral studies, using *t*-tests (details are shown in Table 2).

**Table 2:** Mean ratings, *M* (and standard deviations, *SD*) of judgments on morally offensive and control items on the five dimensions (“This report is plausible”, “This research is convincing”, “This research should be funded”, “This study is well conducted”, “This study provides good evidence regarding the conclusion”). Morally offensive and control items were compared on each dimension, using *t*-tests. Cohen’s *d* indicates the effect size

Dimension	Study	Mean ratings^a^ *M* (*SD*)	*t* values (df = 100)	*p-value*	Effect size *(Cohen’s d)*
This report is plausible	Neutral	*M* = 3.62 *(SD = 0.97)*	3.88	< .001	0.36
	Morally offensive	*M* = 3.25 *(SD* = *1.08)*			
This research is convincing	Neutral	*M* = 3.73 *(SD* = *0.93)*	4.01	< .001	0.38
	Morally offensive	*M* = 3.36 *(SD* = *1.03)*			
This research should be funded	Neutral	*M* = 3.53 *(SD = 1.15)*	2.22	= .029	0.22
	Morally offensive	*M* = 3.28 *(SD = 1.18)*			
This study is well conducted	Neutral	*M* = 3.76 *(SD = 1.00)*	3.38	= .001	0.38
	Morally offensive	*M* = 3.36 *(SD = 1.08)*			
This study provides good evidence regarding the conclusion	Neutral	*M* = 3.71 *(SD = 0.92)*	3.12	=002	0.24
	Morally offensive	*M* = 3.42 *(SD = 1.44)*			

^a^Ratings: 0 = “I do not know”; 1 = “I do not agree at all”; 2 = “I slightly disagree”; 3 = “Neither I agree nor I disagree; 4 = “I slightly agree”; 5 = “I strongly agree”

Ratings on all five dimensions differ significantly between morally offensive and neutral studies. Morally offensive studies compared to neutral studies were rated to be significantly less plausible, significantly less convincing, significantly less worth to be funded, significantly less well conducted, and estimated to provide significantly less strong evidence for the respective conclusion. Hence, Study 1 supports the claim that differences in scientific hypotheses’ perceived moral offensiveness predict differences in explanatory judgments, even when the prior credibility of the hypotheses is controlled for.

One might argue that the effects of the moral offensive content we found in our study were rather small (see effect sizes, indicated by Cohen’s *d* ranging around 0.2), and hence might not greatly affect people’s judgments in everyday life. However, morally offensive content had an effect on all dimensions of explanatory judgment we considered, which indicates that, albeit small, the effect tracks a robust phenomenon. It is not implausible that, in real-life explanatory contexts where reasoning concerns complex subject matters and bodies of evidence, the effect we found subtly interacts with other small biases leading to grossly mistaken explanatory judgments.

In Study 2 we introduced a second possible source of bias. We attempted to replicate the finding of a moral content effect, in the face of a monetary incentive to accurately assess the evidence given by the reports. In particular, we aimed to test whether an incentive to be accurate interacts with the effect of moral content, which we found in Study 1.

## Study 2: Monetary Incentives and Explanatory Judgment

### Participants

A sample of 141 students took part in the experiment, of them 108 students of Tilburg University, The Netherlands (34 male; mean age 19.48, *SD* = 2.22) and 33 students of the University Giessen, Germany (3 male, mean age 24.58 *SD* = 3.06). The students gave written informed consent to participate and received either course credit or a small payment (4 €) for participation. The participants were allocated to either of one group: the “Accuracy” group (*n* = 71, among them 17 participants from Giessen), and the “Control” group (*n* = 70, among them 16 participants from Giessen). All participants gave their informed written consent for participation in the study, in accordance with the Declaration of Helsinki. Participants received careful debriefing prior to the exposure to the experimental material, informing them about the potential offensive character of the material’s content. After the experiment, the experimenter took special care that the participants were aware of the fictitious character of the short reports. The experimental (“accuracy”) group received additional thorough debriefing regarding deceptive claims made by this group’s instruction, after completing the experiment.

Participants of both groups were asked to carefully assess six alleged scientific reports along the same five dimensions as in Study 1. The Accuracy group received an additional instruction. They were explicitly invited to give their ratings as “accurately” as possible. Specifically, they were explained that, for each report, a panel of faculty members and graduate students with relevant expertise had agreed on the most objective (“accurate”) assessments, regarding the five dimensions of interest, in light of the available evidence. Participants of the accuracy group were promised an extra monetary reward in case that their judgments would match these “expert” judgments. Participants were informed about the fact that experts ratings did not exist (and no monetary reward would be paid) only after the experiment during debriefing.

### Materials and Procedure

Study 2 resembled Study 1 but was conducted as a paper and pencil task with the experimental material administered to the participants in suitably prepared booklets. The material was presented in Dutch and in German to the participants in The Netherlands and Germany, respectively. All participants read six short reports about alleged scientific studies, in random orders. Five of the six reports were the same as those in Study 1. The report on children and homosexual families was replaced with a report of an alleged study providing evidence for a positive correlation between a virus and homosexuality. As in study 1, three of the six reports provided evidence for morally offensive hypotheses, and the other three for morally-neutral hypotheses (see Appendix 2 for details).

### Results

For each dimension (“This report is plausible”, “This research is convincing”, “This research should be funded”, “This study is well conducted”, “This study provides good evidence regarding the conclusion”), an ANOVA with the factors Moral (neutral, offensive) × Group (Control, Accuracy) was conducted.

A main effect for Moral was found on the dimension “This report is plausible” ([*F*(1139) = 10.671; *p* = .001; *η* ^*2*^_*part*_ = _._071]). Morally offensive items were judged significantly less plausible than neutral items (*t* (140) = 3.275; *p* = .001; *d* = 0.28). A marginal main effect for Moral was also found on the dimension “This research is convincing” ([*F*(1139) = 3.386; *p* = .068; *η* ^*2*^_*part*_ = _._024]). Morally offensive items were judged marginally significantly less convincing than neutral items (*t* (140) = 1.848; *p* = .067; *d* = 0.19). On the remaining dimensions, morally offensive and morally neutral items did not differ significantly (all *p*s > .40).

A main effect for Group was found on three of the five dimensions: “This research is plausible” [*F*(1139) = 5.048; *p* = .026; *η* ^*2*^_*part*_ = _._035]; “This research should be funded” [*F*(1139) = 5.857; *p* = .017; *η* ^*2*^_*part*_ = _._040], and “This study provides good evidence regarding the conclusion” [*F*(1139) = 5.083; *p* = .026; *η* ^*2*^_*part*_ = _._035]. Judgments in the accuracy group indicated that participants of this group compared to the control group, found the research significantly more plausible (*t* (139) = −2.204; *p* = .029; *d* = 0.20), significantly more worth of being funded (*t* (139) = −2.372; *p* = .019; *d* = 0.20), and providing significantly better evidence for the conclusion (*t* (139) = −2.250; *p* = .026; *d* = 0.20). Judgments on the dimensions “This research is convincing” and “This study is well conducted” did not differ between groups (all *p*s > .20). No interactions between the factors Moral and Group were found (all *p*s > .10). Descriptive statistics are shown in Table 3.

**Table 3:** Mean judgments *M* (and standard deviations, *SD*) for neutral and morally offensive items, rated by participants of the Control and the Accuracy group on the five dimensions: “This report is plausible, “This research is convincing”, “This research should be funded”, “This study is well conducted”, and “This study provides good evidence regarding the conclusion”

	This report is plausible	This research is convincing	This research should be funded	This study is well conducted	This study provides good evidence regarding the conclusion
Control group					
Neutral	*M* = 2.78 (*SD* = 0.88)	*M* = 2.57 (*SD* = 0.90)	*M* = 2.30 (*SD* = 0.85)	*M* = 2.72 (*SD* = 0.93)	*M* = 2.26 (*SD* = 0.88)
Morally offensive	*M* = 2.45 (*SD* = 0.71)	*M* = 2.40 (*SD* = 0.79)	*M* = 2.23 (*SD* = 0.80)	*M* = 2.61 (*SD* = 0.75)	*M* = 2.20 (*SD* = 0.76)
Accuracy group					
Neutral	*M* = 2.99 (*SD* = 0.87)	*M* = 2.72 (*SD* = 0.97)	*M* = 2.54 (*SD* = 0.72)	*M* = 2.70 (*SD* = 0.94)	*M* = 2.41 (*SD* = 0.91)
Morally offensive	*M* = 2.80 (*SD* = 1.00)	*M* = 2.56 (*SD* = 0.97)	*M* = 2.52 (*SD* = 0.87)	*M* = 2.77 (*SD* = 1.06)	*M* = 2.61 (*SD* = 1.05)

The main effects of “Moral” indicate that the moral content of the hypotheses influenced explanatory judgment. The main effects of “Group” indicate that the prospect of receiving a monetary payment influenced explanatory judgments. No interaction effect was found between the factors “Moral” and “Group.” The results support and extend the claim that differences in scientific hypotheses’ perceived moral offensiveness predict differences in explanatory judgments. Further, the prospect of a monetary reward biased participants in favor of higher ratings, although this bias did not further modulate any effect of moral content. Effect sizes for both effects were small, although they systematically biased participants’ judgments on more than one dimension of explanatory judgment. Since moral offense and expectancy of monetary reward are two among the many factors that can impact explanatory reasoning, it is plausible to hypothesize that their influence on reasoning is greater and more intricate in more complex, high-pressure, high-stakes, real-life contexts.

## Discussion. The Psychological Attainability of a Value-Free Science

The present work extends recent research in philosophy of science and in the psychology of explanation by providing direct evidence that explanatory reasoning can be influenced by personal, non-epistemic values. Specifically, the results of our first study support the claim that the perceived moral offensiveness of a scientific hypothesis biases one’s assessment of the evidence for the hypothesis *over and above* the hypothesis’s prior credibility. The results of our second study confirm that explanatory judgments are robustly sensitive to the perceived moral offensiveness of a hypothesis; and they also indicate that a monetary incentive to be accurate in the assessment of the available evidence for an explanatory hypothesis may have no mitigating effect on this bias.

Taken together, our results support three conclusions. First, explanatory judgment is consistently driven by a motivation to avoid inferring a morally undesirable conclusion. Second, the appraisal of the evidential relationship between a particular hypothesis, background beliefs, and a specific body of evidence is affected by moral motivation, and more generally by non-epistemic values. Third, as a matter of psychological fact, the ideal of a value-free science is not attainable. Let us examine each of these conclusions in the light of our studies.

The main effects we found in our two studies can be explained in terms of a motivational bias that affects explanatory judgment. Specifically, the effects can be explained in terms of *morally-motivated explanatory reasoning*: people tend to avoid making certain explanatory judgments based on their negative emotional associations. While it is generally accepted that reasoning processes can be biased to produce emotionally preferable conclusions, psychological research has paid relatively little attention as to whether scientific and explanatory reasoning in particular can be affected by motivational biases, which have been most clearly demonstrated in moral and political reasoning tasks (Ditto et al. 2009).

Given a scientific hypothesis that can explain some available set of data, if differences in the perceived moral offensiveness of the hypothesis systematically predict differences in judgments about the explanatory power of the hypothesis, then it is reasonable to believe that people’s moral values and motivations have a systematic influence on explanatory judgment. This claim would be more persuasive if the correlation between perceived moral offensiveness of the hypothesis and judgments about its explanatory power still obtains once the prior credibility of the hypothesis is controlled for.

Resistance to certain scientific hypotheses can in fact be explained solely in terms of background beliefs: people would refrain from making certain explanatory judgments not because they are motivated to do so by negative emotional associations, but because these judgments are more plausible given people’s prior, background beliefs. For example, one reason why people resist endorsing certain explanatory hypotheses of certain scientific findings is that many of these findings are unnatural and unintuitive, given people’s rich background commonsense understanding of the physical and of the social world (Bloom and Skolnick Weisberg 2007). Another variable that contributes to determine whether people are likely to reject well-confirmed scientific hypotheses seems to be a personality factor such as one’s propensity to endorse any of a number of conspiracy theories (Lewandowsky et al. 2013). So, scientific hypotheses that are inconsistent with our prior, background, commonsense beliefs about the world are likely to be judged as implausible, and may not be endorsed unless they are supported by extra-ordinary evidence gathered by some trustworthy source.

Since our first study controlled for the prior credibility of both the morally-offensive and morally-neutral scientific hypotheses we used, our results cannot be explained solely in terms participants’ background beliefs. To the extent that the perceived moral offensiveness of a scientific hypothesis is associated with negative emotion, our participants avoided making certain explanatory judgments based on their negative emotional association. If this is so, then it can be concluded that our work provides telling evidence for morally-motivated explanatory reasoning.

A second conclusion that can be drawn from our results is that the appraisal of the evidential relationship between a particular hypothesis, background beliefs, and a body of evidence is affected by moral motivation. This morally-motivated appraisal may be due to a partial accumulation of the available evidence, to inaccuracy in aggregating the available evidence with background beliefs concerning the credibility of the hypothesis, or to some error in using all the relevant information available to issue an estimation of the explanatory power of the hypothesis (Kunda 1990).

An alternative account is that moral motivation impacted our participants’ appraisal of the evidential relationship by affording them with different standards of evidence, which they employed to evaluate hypotheses they found to be morally-neutral and hypotheses they found to be morally-offensive (Gilovich 1991; see also Rudner 1953; Douglas 2000). In contrast with morally-neutral hypotheses, when participants evaluated a morally-offensive hypothesis, they could have implicitly employed more stringent evidential standards, asking themselves: “*Must* I believe this?” There is a myriad ways in which a given scientific report—let alone streamlined, alleged reports such as ours—can be criticized, whether because of methodological and statistical flaws, or because of problems in controlling for confounding factors or in drawing correct conclusions from the results. Thus, scientific reasoners that are morally-motivated to disbelieve a hypothesis will easily find some ground for judging a piece of research apparently supporting that hypothesis as ill-conducted, implausible, unconvincing, and inconclusive.

Although our results cannot distinguish which one of these different processes gave rise to the morally-motivated appraisal of the evidence we observed, they show that moral bias may lead one to apparently violate normative models of explanatory judgment based on the rule of Bayesian conditionalization. If this rule is a core normative standard of scientific reasoning (Howson and Urbach 1993) that entails that judgments about the moral value of “believing a proposition cannot supply *new* information about the probability of the proposition” (Sober 2003, p. 117), then our participants apparently violated a core standard of scientific reasoning. The perceived moral offensiveness of the scientific hypotheses supplied our participants with *new* evidence about the hypotheses’ probability, “evidence” that went over and beyond what was warranted by the prior credibility of the hypotheses and by the evidence provided in the scientific reports.

This brings us to the third conclusion underwritten by our results: as a matter of psychological fact, the ideal of a value-free science is not attainable. If reasoners’ assessment of the evidence in support of a scientific hypothesis is systematically affected by judgments about the moral value of the hypothesis, then non-epistemic values systematically intrude the appraisal of the evidential relation, on which the evaluation and justification of scientific claims depend. If scientific reasoning is objective to the extent that the appraisal of scientific hypotheses is insulated from the influence of non-epistemic factors like information about the moral value of scientific hypotheses, then our results provide direct evidence that the value-free ideal is not attainable, as a matter of psychological fact. They also suggest that the notion of objectivity in scientific reasoning should be re-considered.

As the results of our second study indicate, a value-free appraisal of the evidential relation is not easy to promote either. Even when an economic incentive was provided to motivate participants to accurately assess the evidence, we found the same moral bias present as in Study 1. Moreover, participants who expected to receive a monetary reward for accurate judgments were more likely to provide higher ratings of the quality of the research and explanations reported in our mock scientific abstracts.

These findings cohere with Kunda’s (1990, p. 482) observation that it should not be assumed that the motivation to make accurate judgments “will always eliminate biases and improve reasoning. In several studies—she explains—incentives or admonitions to be accurate did not eliminate bias… For accuracy to reduce bias, it is crucial that [participants] possess more appropriate reasoning strategies, view these as superior to other strategies, and be capable of accessing them at will. This is most probably not the case for the biases that have been resistant to accuracy manipulations.” The bias involved in morally-motivated explanatory reasoning may be particularly robust to at least some accuracy manipulations exactly because participants might have serious difficulty in accessing more appropriate explanatory reasoning strategies at will.

One reason why participants may have such a difficulty is that several terms featuring in scientific explanations, such as “virus infection,” “child abuse” and “developmental disorder,” appear to pick out *thick concepts* that possess an ineliminable evaluative dimension (Geertz 1973; Dupré 2007). This evaluative dimension of several scientific explanations is diagnostic of the fact that several scientific hypotheses and results are of particular concern for us, as they are particularly relevant to the satisfaction of our interests and aspirations. Since the truth or falsity of such hypotheses concerns us, we shall find it difficult to employ ordinarily appropriate reasoning strategies to assess them.

In summary, we suggest that the value-free ideal is unrealistic and should be abandoned, since non-epistemic values have influences on scientific judgment that cannot be easily detected and eliminated.

## Conclusions and Questions for Further Research

At least since the 1960s’ an impressive body of research in history, philosophy, and sociology of science has drawn our attention to how interests, values, and cultural and political ideals influence the sciences. Arguments for why the ideal of a value-free science cannot be attainable commonly appeal either to the underdetermination of theory choice by purely epistemic value (e.g., Longino 1990; Solomon 2001) or to the risk of accepting a false theory (or rejecting a true one), which will probably have undesirable non-epistemic consequences (e.g., Douglas 2000; Rudner 1953).^3^ Surprisingly, little attention has been paid to relevant evidence about reasoning and valuing in the psychological literature.

Bringing psychological results to bear on the debate about objectivity in scientific reasoning and the ideal of a value-free science, our results give substance to a third reason why the ideal of a value-free science may not be attainable. As a matter of psychological fact, for several scientific hypotheses, non-epistemic values—including political, moral, and religious values—tend to bias the appraisal of the evidential relation, and lead reasoners to make explanatory judgments that do not accord with core normative standards of explanatory reasoning. If explanatory judgment is taken to be objective to the extent that the appraisal of scientific hypotheses is not influenced by the degree to which the hypotheses are found morally offensive, but only by the available evidence and the prior credibility of the hypotheses, then non-objectivity may be characteristic of explanatory judgments about a large class of scientific hypotheses.

Our conclusions motivate three sets of questions for further research. First, how can the effects on explanatory judgment of hypotheses’ perceived moral offensiveness be further disentangled from the effects of the hypotheses’ prior credibility? Second, does expertise have a mitigating effect on the impact of the perceived moral offensiveness of a hypothesis on explanatory judgments about the hypothesis? Third, how does the social character of scientific inquiry affect morally-motivated explanatory reasoning? Let us conclude by considering each question in turn.

First, our studies disentangled hypotheses’ perceived moral offensiveness and prior credibility by asking a group of participants, different from those involved in Studies 1 and 2, to rate a number of statements. A different approach would be to start off with morally neutral hypotheses that all participants assess for prior credibility, then inform only half of the participants of morally problematic consequences or associations of the hypotheses, and finally present all participants with the same scientific reports and ask for their explanatory judgments. If our conclusions about morally-motivated explanatory reasoning are correct, then explanatory judgments should be more negative in the group given the information about the objectionable associations, even when we control for prior credibility assessments.

Second, the participants in our studies were educated laypersons, not scientists. But it is scientists that have gone through years of training to be more careful reasoners regarding scientific hypotheses and reports falling within their areas of expertise; and it is scientists’ reasoning and explanatory judgment that are relevant to questions concerning the ideal of value-free science and objectivity in scientific reasoning. One may thus expect that professional scientists do not suffer from the same judgmental biases that we have demonstrated in undergraduates and educated laypersons. So, our results, it can be held, directly bear on how laypeople understand scientific information, but not immediately on issues in philosophy of science.

However, psychological research in both naturalistic and laboratory settings has demonstrated that a wide variety of biases often affect assessments that many professionals, including physicians, investors, accountants, option traders, real estate agents, engineers, psychologists and philosophers, are trained to make (e.g., McNeil et al. 1982; Choi and Pritchard 2003; Bazerman et al. 2002; Fox et al. 1996; Buckwalter 2014; Schwitzgebel and Cushman 2012, 2015). Though expertise in specific domains has obvious positive effects on judgment and decision-making, these findings indicate that experienced professionals often “display either roughly the same biases as college students or the same biases at somewhat reduced levels” (Plous 1993, p. 258). So, it is not implausible to hypothesise that our conclusions about morally-motivated explanatory reasoning may sometimes apply to the reasoning of many professional scientists too.

Third and finally, our studies concentrated on individuals’ explanatory judgment. But science is a social practice, and some have argued that the ideal of a value-free science should not be tied to individuals’ freedom from various kinds of bias; rather, it should be related to the social character of inquiry and secured through scientists’ open discourse and criticism (e.g., Longino 1990; Popper 1934). From this perspective, objectivity in scientific reasoning would result from an intersubjective process in which individuals’ judgments are mutually, critically and openly assessed.

While these claims can be assessed for their psychological plausibility, it is not obvious when and to what extent the social character of scientific inquiry would control for detrimental effects of morally-motivated individual explanatory reasoning. Collaborative efforts can sometimes improve individual judgments and reasoning, leading groups to outperform their individual members (Doris and Nichols 2012). However, group reasoning can also bring about outcomes worse than the outcomes that members could have brought about individually (e.g., Bahrami et al. 2010; see also Skoyles 2010). Groups are known to be prone to a number of psychological biases such as in-group/out-group bias (Sherif 1966), emotional contagion (Hatfield et al. 1993), “group-think” (Janis 1972), and also to conformism even in the moral domain (Lisciandra et al. 2013). Similarly to the field of social psychology, all scientific communities may well be biased towards politically or morally homogenous views that pose a threat to good science and sound scientific reasoning (Duarte et al. 2015). For psychologists and philosophers alike, it therefore remains an exciting project to investigate how different kinds of social contexts impact scientific reasoning.

## Appendices

### Appendix 1. Material for Pre-Evaluation (Translated from Dutch)

1. Reproducing during a high tide increases the chances of giving birth to a female.
2. Homosexuality can be caused by infection.
3. Developmental disorders occurs more often in children with parents of the same gender.
4. Going to bed late increases the chances of becoming a millionaire.
5. Long sleepers have a bigger chance of being engaged in sexual abuse than children.
6. Full moon increases the chances of being fatigued at night.
7. Being a male increases the chances of being professionally satisfied and successful in life
8. Eating pizza regularly every day increases the chances of immunity to flu.
9. Men are more successful than women because they are more motivated and they have more cognitive capacities.
10. Living in a windy place increases the chances of possessing a bicycle.
11. Being infected with the Merrosa-virus increases the chances of being homosexual in mammals.
12. Owning a dog increases the chances of winning the lottery.
13. Getting up late increases the chances of committing child abuse.
14. Attending religious service frequently, at least once a week, increases the chances of maintaining a good health.
15. Living in a place where beer price is lower than the national standard increases the chances of risky sexual behavior.
16. Consuming breakfast regularly every morning increases the chances of having a healthy body mass index
17. There is a significant correlation between attending religious services and lower chances of death.
18. Consuming breakfast makes girls thinner.

### Appendix 2. Material for Study 1 and 2

#### Eating Pizza Regularly Every Day Increases the Chances of Immunity to Flu

(Used in study 1 and study 2)

A recent study conducted by French researchers investigated the link between eating pizza and immunity to flu. The researchers monitored 1461 participants aged between 12 and 88 every 3 months for a period of 6 years. Participants’ life style, health conditions and diet were the focus of research. Specifically, along with blood and urine tests, samples from the back of all participants’ nose and throat were taken. Among the 78 participants who consumed pizza Napoletana regularly on a daily basis, 98 % showed significantly higher immunity to flu virus. Among the 1383 participants who did not eat pizza Napoletana on a daily basis, only 1.1 % showed significantly higher immunity to flu virus. Factors such as family health history, age, sex or personal income could not account for the results. The study therefore supports the hypothesis that eating pizza regularly, every day, increases the chances of being immune to flu virus.

#### Attending Religious Service Frequently, at Least Once a Week, Increases the Chances of Maintaining a Good Health

(Used in study 1 and study 2)

A recent study conducted by British researchers investigated the link between regular attendance at religious services and the individual state of health. The researchers surveyed 3815 individuals aged between 40 and 69 about their religious behavior, such as the frequency of religious service attendance, and collected longitudinal data from general, regular health check-ups over a period of 20 years. Among the 1826 participants who attended religious services at least once a week, 68 % obtained a positive result on all the health check-ups. Among the 1989 participants who did not attend religious services in the last month, only 28 % obtained a positive result in all the health check-ups. Factors such as family health history, age, sex or personal income could not account for the results. The study therefore supports that attending religious services at least once a week increases the chances of maintaining a good health.

#### Being a Male Increases the Chances of Being Professionally Satisfied and Successful in Life

(Used in study 1 and study 2)

A recent study conducted by American researchers investigated the link between gender and professional success. Across 20 years, the researchers observed 4239 young adults with high learning abilities, with regard to their professional attitudes and achievements in life. Among the 2215 male participants, 78 % earned a PhD degree at some point. Comparing their performance to the goals set by their company, male participants showed higher rates of success; they also showed higher rates of professional satisfaction. Among the 2024 female participants, only 34 % earned a PhD degree at some point. Comparing their performance to the goals set by their company, female participants showed lower rates of success; they also showed lower rates of professional satisfaction. Factors such as marital status, type of occupation, and leisure time activities could not account for the result. The study therefore supports the hypothesis that being a male increases the chances of being professionally satisfied and successful in life.

#### Getting up Late Increases the Chances of Being Convicted for Child Abuse

(Used in study 1 and study 2)

A recent study of Australian researchers investigated the link between getting up late in the morning and being convicted for child abuse. The researchers surveyed 7200 participants from Australia, New Zealand, Germany, and the Netherlands, regarding their sleeping habits and waking times during 30 years. Among the 1900 participants who persistently went to bed after midnight and rose after 11 am, 9 % were at least once convicted for physical, sexual or emotional maltreatment or neglect of a child or children. Among the 5300 participants who persistently went to bed before midnight and rose before 11 am, only 0.7 % were convicted for physical, sexual or emotional maltreatment or neglect of a child or children. Factors such as personal criminal record, mental health status, or one’s overall amount of sleep could not account for the result. The study therefore supports the hypothesis that getting up late in the morning increases the chances of being convicted for child abuse.

#### Consuming Breakfast Regularly Every Morning Increases the Chances of Having a Healthy Body Mass Index

(Used in study 1 and study 2)

A recent study conducted by Italian researchers investigated the link between regular consumption of breakfast and a healthy Body Mass Index (BMI). The researchers surveyed 4474 girls, aged 18 and 19 about their breakfast consumption and assessed their BMI. Among the 2181 girls who consumed breakfast regularly, 73 % were assessed to have a healthy BMI. Among the 2293 girls, who had not consumed breakfast regularly, only 51 % had a healthy BMI. Factors such as the social and economic background, genetic differences, or differences in daily physical activity could not account for the results. The study therefore supports the hypothesis that regular breakfast consumption increases the chances of having a healthy BMI.

#### Being Raised by Parents of the Same Sex Increases the Chances of Suffering from Developmental Disorders

(Used in study 1)

A recent study conducted by Australian researchers investigated the link between being raised by homosexual parents and suffering from developmental disorders. The researchers screened 1687 children aged between 3 and 12 for disorders of psychological development, specifically for symptoms of autistic spectrum disorder (ASD). Among the 185 children raised by parents of the same sex, 3.4 % were diagnosed autistic disorder, or some other disorder of language or learning. Among the 1502 children raised by a heterosexual couple, only the 0,3 % were diagnosed autistic disorder, or some other disorder of language or learning. Factors such as parents’ socio-economic status, the children’s genetic history, and level of stress in infancy could not account for the results. The study therefore supports the hypothesis that being raised by a homosexual family increases the chances of suffering from developmental disorders.

#### Virus Infection Increases the Chances of Being Homosexual

(Used in study 2)

A recent study of Canadian researchers investigated the link between virus infection and homosexuality in mammals. The researchers focused on sheep (Ovis aries) and on Japanese macaque monkeys (Macaca fuscata). They screened 3731 sheep and 72 macaques for Merrosa-virus infection, which may be involved in homosexual orientation in mammals. Then, the researchers studied all the animals during breeding season regarding their sexual orientation, and sexual behavior. Among the 938 sheep identified as being infected with the virus, 87 % were found to have homosexual orientation. Among the 2793 sheep identified as being free from the virus, only 0.8 % were found to have homosexual orientation. Among the 22 macaques identified as being infected with the virus, 90 % were found to have homosexual orientation. Among the 50 macaques identified as being free from the virus, only 1 % were found to have homosexual orientation. Factors such as epigenetic and developmental differences, or age and sex of the animals could not account for the results. This study therefore supports the hypothesis that being infected with the Merrosa-virus increases the chances of being homosexual.

## Footnotes

1 Prominent among other languages were Marathi, Hindi, and Tamil. It should be noted that India, where those languages are spoken, is a country with a highly stratified and patriarchic social system It is an open question to what extent the results we found in this study were driven by some idiosyncratic cultural component.

2 It is important to clarify that the first and second items in this list aimed at eliciting judgments about the degree to which the reports were found authentic (i.e., non-bogus). Instead, the credibility ratings collected in the pre-study uncovered the prior, subjective credibility of the individual hypotheses discussed in the reports.

3 Richard Jeffrey (1956) denies the assumption that “no analysis of what constitutes the method of science would be satisfactory unless it comprised some assertion to the effect that the scientist as scientist *accepts or rejects* hypotheses” (Rudner 1953, p. 2). According to Jeffrey, scientists as scientists are not concerned with accepting and rejecting hypotheses, but only with gathering and assessing the evidence so as to assign probabilities to hypotheses.

## References

[CR1] Bahrami B, Olsen K, Latham PE, Roepstorff A, Rees G, Frith CD (2010). Optimally interacting minds. Science.

[CR2] Balcetis E, Dunning D (2006). See what you want to see: Motivational influences on visual perception. Journal of Personality and Social Psychology.

[CR3] Bazerman MH, Loewenstein G, Moore DA (2002). Why good accountants do bad audits. Harvard Business Review.

[CR4] Bloom P, Skolnick Weisberg D (2007). Childhood origins of adult resistance to science. Science.

[CR5] Buckwalter, W. 2014. Intuition fail: Philosophical activity and the limits of expertise. *Philosophy and Phenomenological Research*. doi:10.1111/phpr.12147.

[CR6] Choi SJ, Pritchard AC (2003). Behavioral economics and the SEC. Stanford Law Review.

[CR7] Ditto PH, Lopez DF (1992). Motivated skepticism: Use of differential decision criteria for preferred and nonpreferred conclusions. Journal of Personality and Social Psychology.

[CR8] Ditto, P.H., D.A Pizarro, and D. Tannenbaum. 2009. Motivated moral reasoning. In *The psychology of learning and motivation*, eds. D.M. Bartels, C.W. Bauman, L.J. Skitka, and D.L. Medin, 50: 307–338. Burlington VT: Academic Press.

[CR9] Dorato M, Machamer P, Wolters G (2004). Epistemic and non-epistemic values in science. Science, values and objectivity.

[CR10] Doris JM, Nichols S, Margolis E, Samuels R, Stich S (2012). Broadminded: Sociality and the cognitive science of morality. The Oxford handbook of philosophy and cognitive science.

[CR11] Douglas H (2000). Inductive risk and values in science. Philosophy of Science.

[CR12] Douglas H (2009). Science, policy, and the value-free ideal.

[CR13] Duarte, J.L., Crawford, J.T., Stern, C., Haidt, J., Jussim, L., and P.E. Tetlock. 2015. Ideological diversity will improve social psychological science. *Behavioral and Brain Sciences*. 10.1017/S0140525X14000430 25036715

[CR14] Dupré, J. 2007. Fact and value. In *Value free science: ideal or illusion?*, eds. Harold Kincaid, John Dupré, Alison Wylie, 21–41. Oxford: Oxford University Press.

[CR15] Elliott KC (2011). Is a little pollution good for you? Incorporating societal values in environmental research.

[CR16] Elliott KC, Resnik DB (2014). Science, policy, and the transparency of values. Environmental Health Perspectives.

[CR17] Fox CR, Rogers BA, Tversky A (1996). Options traders exhibit subadditive decision weights. Journal of Risk and Uncertainty.

[CR18] Geertz C (1973). Interpretation of cultures.

[CR19] Gilovich T (1991). How we know what isn’t so: The fallibility of human reason in everyday life.

[CR20] Greenhoot AF, Semb G, Colombo J, Schreiber T (2004). Prior beliefs and methodological concepts in scientific reasoning. Applied Cognitive Psychology.

[CR21] Haack S (1993). Knowledge and propaganda: Reflections of an old feminist. Reason Papers.

[CR22] Harris AJL, Corner A, Hahn U (2009). Estimating the probability of negative events. Cognition.

[CR23] Hatfield E, Cacioppo JT, Rapson RL (1993). Emotional contagion. Current Directions in Psychological Science.

[CR24] Howson C, Urbach P (1993). Scientific reasoning: The Bayesian approach.

[CR25] Janis IL (1972). Victims of groupthink: A psychological study of foreign-policy decisions and fiascoes.

[CR26] Jeffrey R (1956). Valuation and acceptance of scientific hypotheses. Philosophy of Science.

[CR27] Koehler J (1993). The influence of prior beliefs on scientific judgments of evidence quality. Organizational Behavior and Human Decision Processes.

[CR28] Krizan Z, Windschitl PD (2007). The influence of outcome desirability on optimism. Psychological Bulletin.

[CR29] Kuhn, T.S. 1977. Objectivity, value judgment, and theory choice. In *The essential tension: Selected studies in scientific tradition and change*, ed. T.S. Kuhn, 320–339. Chicago: University of Chicago Press.

[CR30] Kunda Z (1987). Motivation and inference: Self-serving generation and evaluation of evidence. Journal of Personality and Social Psychology.

[CR31] Kunda Z (1990). The case for motivated reasoning. Psychological Bulletin.

[CR32] Laudan L (1984). Science and values.

[CR33] Lewandowsky S, Oberauer K, Gignac GE (2013). NASA faked the moon landing —therefore, (climate) science is a hoax an anatomy of the motivated rejection of science. Psychological Science.

[CR34] Lisciandra C, Postma-Nilsenová M, Colombo M (2013). Conformorality. A study on group conditioning of normative judgment. Review of Philosophy and Psychology.

[CR35] Longino H (1990). Science as social knowledge.

[CR36] Longino H, Hankinson Nelson L, Nelson J (1996). Cognitive and non-cognitive values in science: Rethinking the dichotomy. Feminism, science, and the philosophy of science.

[CR37] Lord, C.G., L. Ross, and M.R. Lepper. 1979. Biased assimilation and attitude polarization: the effects of prior theories on subsequently considered evidence. *Journal of Personality and Social Psychology* 37(11): 2098–2109.

[CR38] MacCoun RJ (1998). Biases in the interpretation and use of research results. Annual Review of Psychology.

[CR39] Machamer P, Wolters G, Machamer P, Wolters G (2004). Introduction: Science, values and objectivity. Science, values and objectivity.

[CR40] McNeil BJ, Pauker SG, Sox HC, Tversky A (1982). On the elicitation of preferences for alternative therapies. The New England Journal of Medicine.

[CR41] Norton MI, Vandello JA, Darley JM (2004). Casuistry and social category bias. Journal of Personality and Social Psychology.

[CR42] Plous S (1993). The psychology of judgment and decision making.

[CR43] Popper, K.R. 1934/2002. *Logik der Forschung*, Berlin: Akademie Verlag. English translation as *The Logic of Scientific Discovery*, London: Routledge.

[CR44] Pyszczynski T, Greenberg J, Holt K (1985). Maintaining consistency between self-serving beliefs and available data: A bias in information evaluation. Personality and Social Psychology Bulletin.

[CR45] Reiss, J., and J. Sprenger. 2014. Scientific objectivity. In *The stanford encyclopedia of philosophy (Fall 2014 Edition)* ed. E.N. Zalta. http://plato.stanford.edu/archives/fall2014/entries/scientificobjectivity.

[CR46] Resnik DB (2007). The price of truth: How money affects the norms of science.

[CR47] Rudner R (1953). The scientist qua scientist makes value judgments. Philosophy of Science.

[CR48] Ruphy S (2006). ‘Empiricism all the way down’: A defense of the value-neutrality of science in response to Helen Longino’s contextual empiricism. Perspectives on Science.

[CR49] Schwitzgebel E, Cushman FA (2012). Expertise in moral reasoning? Order effects on moral judgment in professional philosophers and non-philosophers. Mind & Language.

[CR50] Schwitzgebel E, Cushman FA (2015). Philosophers’ biased judgments persist despite training, expertise and reflection. Cognition.

[CR51] Sherif M (1966). In common predicament: Social psychology of intergroup conflict and cooperation.

[CR52] Skoyles JR (2010). Optimizing scientific reasoning. Science.

[CR53] Sober E, Kinkaid H, Dupré J, Wylie A (2007). Evidence and value freedom. Value-free science —Ideal or illusion?.

[CR54] Solomon M (2001). Social empiricism.

[CR55] Uhlmann EL, Cohen GL (2005). Constructed criteria: Redefining merit to justify discrimination. Psychological Science.

[CR56] Wyer RS, Frey D (1983). The effects of feedback about self and others on the recall and judgments of feedback-relevant information. Journal of Experimental Social Psychology.

